# A New Artificial Intelligence Approach Using Extreme Learning Machine as the Potentially Effective Model to Predict and Analyze the Diagnosis of Anemia

**DOI:** 10.3390/healthcare11050697

**Published:** 2023-02-26

**Authors:** Dimas Chaerul Ekty Saputra, Khamron Sunat, Tri Ratnaningsih

**Affiliations:** 1 Department of Computer Science and Information Technology, College of Computing, Khon Kaen University, Khon Kaen 40000, Thailand; 2 Department of Clinical Pathology and Laboratory Medicine, Faculty of Medicine, Public Health and Nursing, Universitas Gadjah Mada, Yogyakarta 55281, Indonesia

**Keywords:** anemia, extreme learning machine, beta thalassemia trait, iron deficiency anemia, hemoglobin E, complete blood count

## Abstract

The procedure to diagnose anemia is time-consuming and resource-intensive due to the existence of a multitude of symptoms that can be felt physically or seen visually. Anemia also has several forms, which can be distinguished based on several characteristics. It is possible to diagnose anemia through a quick, affordable, and easily accessible laboratory test known as the complete blood count (CBC), but the method cannot directly identify different kinds of anemia. Therefore, further tests are required to establish a gold standard for the type of anemia in a patient. These tests are uncommon in settings that offer healthcare on a smaller scale because they require expensive equipment. Moreover, it is also difficult to discern between beta thalassemia trait (BTT), iron deficiency anemia (IDA), hemoglobin E (HbE), and combination anemias despite the presence of multiple red blood cell (RBC) formulas and indices with differing optimal cutoff values. This is due to the existence of several varieties of anemia in individuals, making it difficult to distinguish between BTT, IDA, HbE, and combinations. Therefore, a more precise and automated prediction model is proposed to distinguish these four types to accelerate the identification process for doctors. Historical data were retrieved from the Laboratory of the Department of Clinical Pathology and Laboratory Medicine, Faculty of Medicine, Public Health, and Nursing, Universitas Gadjah Mada, Yogyakarta, Indonesia for this purpose. Furthermore, the model was developed using the algorithm for the extreme learning machine (ELM). This was followed by the measurement of the performance using the confusion matrix and 190 data representing the four classes, and the results showed 99.21% accuracy, 98.44% sensitivity, 99.30% precision, and an F1 score of 98.84%.

## 1. Introduction

The main task of the circulatory system is to allow the flow of blood, oxygen, and nutrients to all cells and tissues in the body [1,2]. However, there are disorders of the circulatory system, better known as blood disorders, in which blood circulation is obstructed [3,4] Blood disorders commonly experienced by humans include anemia, hemophilia [5,6], and blood clots [7,8], as well as blood cancers such as leukemia [9,10], lymphoma [11,12], and myeloma [13,14]. A blood disorder is a condition that affects the ability of blood to function properly in humans [15], with most of these disorders having the capacity to reduce the number of cells, proteins, platelets, or nutrients in the blood, thereby impairing its function [16,17]. It is important to note that most of these problems are caused by abnormalities in certain genes and can be passed down via families [18]. Some medical issues such as drug use and lifestyle also lead to blood abnormalities [19]. It has been reported that anemia is the most common blood disorder seen in humans [20].

**Figure 1:**
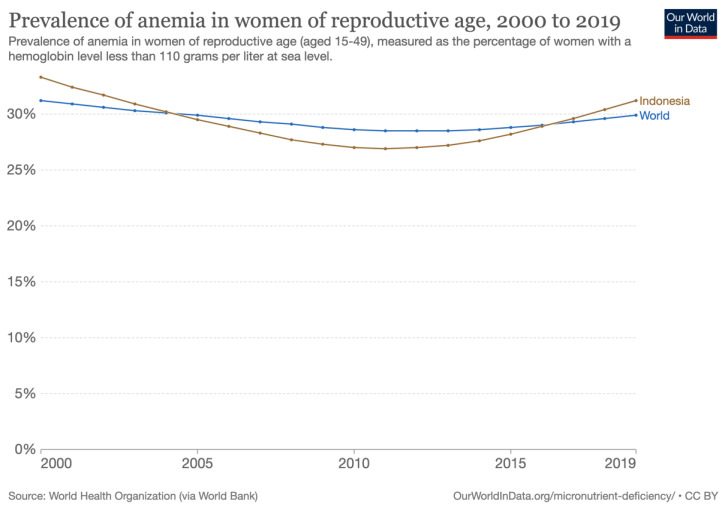
Adapted from Ref. [32] about prevalence of anemia in Indonesia and worldwide: A case problem in women of reproductive age, 2000 to 2019. Copyright (2019) World Health Organization (via World Bank).

Anemia has been defined as a decrease in red blood cells, hemoglobin, and the blood’s ability to carry oxygen throughout the body [21,22]. It is a serious and persistent issue affecting individuals worldwide [23,24]. The prevalence of anemia among Indonesian women of reproductive age is shown in Figure 1 to exceed the global incidence [25]. A previous study also noted that iron deficiency is the major cause of anemia in every part of the globe [26]. As previously stated, this disease is the substantial reduction in the number of red blood cells circulating inside the body [27], thereby leading to a great decrease in the ability of the blood to transport oxygen [28]. The diagnosis of anemia is usually confirmed by the concentration of hemoglobin in the blood or the hematocrit, which is the ratio of the number of red blood cells to the total volume of a blood sample [29]. A patient with hemoglobin or hematocrit values that are more than two standard deviations below the normal range is believed to have anemia [30]. Meanwhile, the blood’s hemoglobin and hematocrit levels may not adequately reflect the severity of the anemia in a patient with a low RBC mass who is also suffering from hypovolemia-caused dehydration-induced plasma volume loss because the values are likely to fall within the normal range [31].

There are many people suffering from anemia in Indonesia [33]. Iron is believed to be an essential component of several enzymes; it has a role in the formation of hemoglobin in the human body [34]. This means its deficiency can cause anemia [35]. A survey conducted showed that the frequency of anemia in the Indonesian population was anticipated to grow by 0.8% in 2019 reaching 31.20% of the population [32]. The prevalence of anemia sufferers worldwide increased by 0.3% in 2019 to 29.90% [32]. It has also been noted that abnormal production of alpha (α)- or beta (β)-globin chains is the root cause of thalassemia, which is a hematological disease that runs in families, usually passed down from one generation to another [36,37]. Serum ferritin levels [38], serum iron [39], total iron binding capacity, and transferrin saturation percentage are the tests most often used to confirm the presence of IDA, as presented in Table 1 [40,41]. The identification of BTT and HbE is normally performed through Hb tests using high-performance liquid chromatography, capillary/hemoglobin electrophoresis, or DNA analysis [42,43]. The application of DNA analysis is not accessible in normal labs due to the need for specialized equipment; in addition, it is time-consuming and expensive [44]. When a patient is assumed to be anemic, doctors often prescribe a hematology test cassette that includes the diagnosis [45]. The high cost of this laboratory test can exhaust patients’ resources and government funds, thereby precipitating a financial crisis in the national healthcare systems of low- and middle-income nations. Therefore, a web-based application is proposed in this study to assist doctors in prescribing cost-effective and sensible laboratory tests for the diagnosis of anemia to aid in the rational use of laboratories by clinicians.

**Figure 2:**
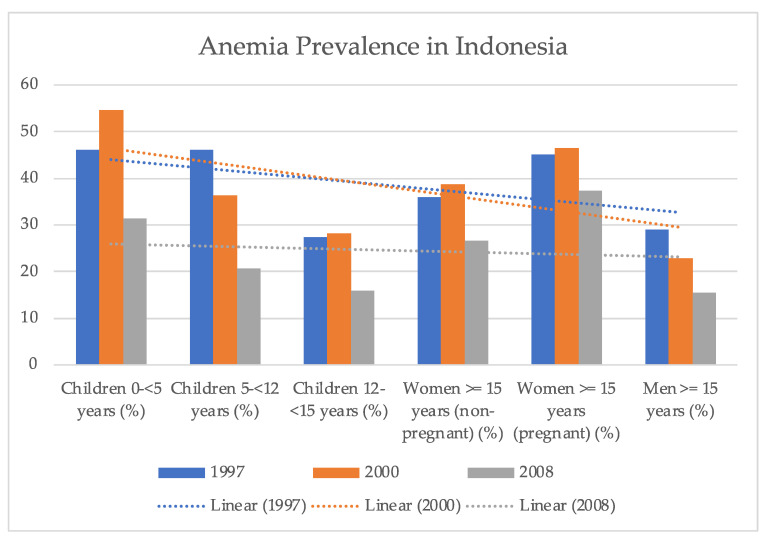
Adapted from Ref. [47] about during the second, third, and fourth waves of the Indonesia Family Life Surveys (IFLS), the prevalence of anemia among children, women, and men were evaluated. These waves occurred from 2005 to 2008. Copyright (2015), Jonathan S Barkley, Katherine L Kendrick, Karen Codling, Siti Muslimatun, and Helena Pachón.

Anemia is a significant threat to public health on a global scale, and its incidence is disproportionately higher among young children and pregnant women. According to the World Health Organization (WHO), 42% of children under the age of five and 40% of pregnant women worldwide are affected by anemia [46]. Indonesia Family Life Surveys (IFLS) reported that the prevalence among Indonesian children, adolescents, women, and men continued to fall from 1997 to 2008 as indicated in Figure 2 [47]. The anticipated decline among non-pregnant women in Southeast Asia between 1995 and 2011 was predicted to be 8% less than the 9.4% decline among non-pregnant women between 1997 and 2008 as reported by IFLS [47]. It was also discovered that the reduction among pregnant women during the period was comparable to IFLS (7.8%) and Southeast Asian (9%) estimates [47]. However, the decline among children under the age of five was much greater in the IFLS (14.6%) than in Southeast Asia (4%) [47].

The Fick equation is normally used to determine the flow of oxygen to a certain bodily region [48] using three independent variables, including the blood flow [49], the concentration of red blood cells [50], and the portion of hemoglobin that has released oxygen on its journey from the arteries to the veins [51]. The oxygen-carrying capacity of anemic individuals’ blood was discovered to be diminishing while the remaining two variables underwent compensatory modifications as illustrated in Equation (1) and in the further discussion in [52].

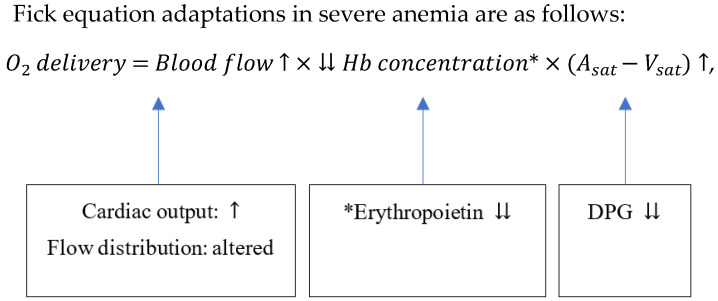
(1)
where DPG is diphosphoglycerate, $A_{sat}$ is arterial blood, and $V_{sat}$ is venous blood.

In anemic individuals, the blood flow to crucial organs such as the heart, brain, liver, and kidneys is boosted, while the blood supply to less vital organs is diminished [53]. The anemic patient appears pale because blood is taken from the skin to ensure the vital organs continue to get sufficient oxygen [54]. Moreover, cardiac output is projected to be lower at rest and greater during exercise in individuals with mild or severe anemia compared to healthy individuals [55]. It has also been reported that severe anemia has the ability to increase resting cardiac output in individuals with coronary artery disease or other preexisting cardiovascular diseases, thereby increasing their risk of developing high-output heart failure [55].

It was discovered, as shown in Table 1, that indices and discriminant formulas had promising prediction outcomes in several investigations, but the forecast findings for different populations remain unsatisfactory, particularly when assessing the efficacy of the methodologies used. Some research was observed to be contentious due to gender, age, or ethnic variances [56,57,58]. Meanwhile, machine learning-based strategies are believed to have a short-term translational effect. This is indicated by their most significant applications in the field of biomedicine such as medical diagnostics, radiological diagnostics, and medication synthesis. Therefore, it is feasible to create discriminant models using machine learning software approaches to undertake large-scale assessments of laboratory data. It is pertinent to state that machine learning is one of the subfields under the umbrella of artificial intelligence [59].

Artificial intelligence (AI) is defined as the effort of computers to simulate human cognitive processes [60]. This is observed in signal processing, voice recognition, expert systems, and natural language processing. The continuous development of interest in AI has enhanced competition among businesses to showcase the AI features of their goods and services [61]. It is important to note that the creation and training of machine learning and deep learning algorithms require the application of specialized hardware and software [62]. This is mainly due to the fact that the operation of AI systems entails the intake of massive quantities of labeled training data as well as the analysis of the data for correlations and patterns to predict future states [61]. AI programming focuses on three cognitive skills, which include learning [59], reasoning [63], and self-correction [64].

This aspect of AI programming involves the collection of data and the development of rules to translate the data into actionable knowledge. Algorithms provide computer systems with detailed instructions to perform a certain task [65]. It is important to state that AI technology is experiencing a time of rapid expansion, but few are aware it has several subfields, one of which is deep learning [65]. The subfields and branches were developed to reduce the very large scope of AI for the purpose of development or research [66]. It is anticipated that AI has the ability to expedite the process of discovering human issues. For example, Laengsri et al. [67] classified 6935 data obtained from the Medical Laboratory Service Centre, Faculty of Medical Technology, Mahidol University between July 2014 and September 2016 as either thalassemia trait or iron deficiency anemia using k-nearest neighbor, decision tree, random forest, artificial neural network, and support vector machine methods. It was discovered that the decision tree algorithm attained a maximum degree of accuracy of 98.03%. The study used seven hematology analyzer-generated features to determine the existence of anemia in individuals. This is necessary because it is difficult to determine the kind of anemia present in a patient using only a blood sample. The findings are expected to allow medical personnel to conduct further diagnostic tests without difficulty and also to ensure a more precise and specific diagnosis [67].

Another study also applied extreme learning machines and regularized extreme learning machines to anemia cases [58]. A total of 342 patients, including 152 with beta-thalassemia-type anemia obtained from the Elazig Public Health Laboratory between 1 December 2016 and 23 May 2019 and 190 with iron deficiency anemia obtained from the Elazig City Hospital Biochemistry Laboratory between 1 March 2018 and 31 July 2018, were studied. The investigation considered a large number of other variables such as gender, in addition to the findings of the clinical pathology test. The regularized extreme learning machine approach produced a 95.59%accuracy rate by combining the k-nearest neighbor, support vector machine, extreme learning machine, and regularized extreme learning machine methods [58].

This present research focuses on establishing an AI model to rapidly, precisely, and reliably diagnose anemia. The process involves classifying anemia into four types, which include beta thalassemia trait (BTT), hemoglobin E (HbE), iron deficiency anemia (IDA), and combination (BTT and IDA or HbE and IDA) using the extreme learning machine approach. Previous studies [23,52,57,66] have shown the relevance of data mining and increasing computing capacity in several biological applications. Therefore, this research seeks to develop a trustworthy and interpretable computational model through the following: (a) collection of clear and dependable laboratory datasets for training and validation, (b) demonstration of dataset characteristics or descriptors to predict the intrinsic properties, and (c) development of a simple and interpretable model.

## 2. Related Work

Support vector machines (SVM), naive Bayes (NB), decision trees (DT), k-nearest neighbor (KNN), multilayer perceptron (MLP), hybrid classifier machine learning, average ensemble (AE), genetic algorithm convolutional neural network (GA-CNN), genetic algorithm stacked-encoder (GA-SAE), support vector machines (SVM), and random forest (RF) are different types of AI. Several studies have been published on the application of machine learning to categorize different kinds of anemia, as indicated by [68,69,70], which forecasted data in the form of a complete blood count (CBC) and constructed a model to identify anemia.

Hemoglobin level estimation is an important step in any task related to blood analysis [50], and it also determines whether a person is anemic. A study [68] used blood test characteristics and applied a machine learning model to calculate hemoglobin levels and identify anemia. The dataset used consists of 9004 data with 75%, or 6753, for training and 25%, or 2251, for testing. A total of three machine learning algorithms—including DT, NB, and NN, as well as a combination of all three approaches known as a hybrid classifier—were applied. Moreover, the MAE and RMSE methodologies were used to assess the performance of the approach, and the MAE results showed that the hybrid classifier had 0.083, the best RMSE value of 0.015, and an accuracy of 0.996% [68].

Tremendous advances in the healthcare industry have resulted in the production of significant data in everyday life [71]. There is a need to extract information for analysis, prediction, recommendation, and decision-making purposes. It was discovered in the realm of medical research that the prediction of disease is essential to design effective prevention and treatment methods. The presentation of wrong information occasionally leads to death. Therefore, a recent study applied 200 CBC data fields obtained from the Pathology Centre and Laboratory Test Centre, as well as RF, C4.5, and NB, which are considered three distinct types of machine learning. K-fold cross-validation and mean absolute error were both used at different stages of the model evaluation process. It was discovered that the C4.5 approach produced the most precise answers, with a precision percentage of 96.0909 and an absolute mean error of 0.0333 [70].

Anemia was also found to be a severe public health problem, particularly for children, in Bangladesh [69]. Thus, the prediction of illness is essential to formulate community and healthcare policy as well as to forecast resource planning. The study used the common risk variables to determine the appropriate machine learning method to predict anemia status in children (under five years) [69]. The 2013 data containing all relevant characteristics for the children, obtained through a nationally representative cross-sectional study conducted by the Bangladesh Demographic and Health Survey (BDHS) in 2011, were used. The investigation employed six techniques, which included the LDA, CART, KNN, SVM, RF, and LR, and they were assessed using the confusion matrix, accuracy, sensitivity, and specificity. The findings showed that the CART approach yielded the greatest evaluation scores of 62.35%, 71.54%, and 53.52% [69].

It is important to note that “deep learning” and “machine learning” are interchangeable when discussing artificial intelligence (AI) [62]. Deep learning is established based on the concept of creating learning algorithms or models that can simulate the human brain [65]. Humans use neurons in their brains to process information, while deep learning algorithms utilize artificial neural networks to perform the same function [72]. Some recent studies [73,74,75] used deep learning to enhance the process of identifying anemia in patients. The single red blood cell count imaging data of 130 individuals with sickle cell anemia (SCA) were surveyed and discovered to exceed 9000 single red blood count image data of patients [73]. SCA is a severe hematological illness that often leads to lifelong hospitalization and, in some circumstances, death [73]. It is important to note that the manual location and classification of aberrant cells in the blood films of SCA patients is time-consuming, difficult, and error-prone, and it requires the skill of a hematologist. The study used the AlexNet deep learning model, and the accuracy was recorded to be 95.92%, sensitivity was 77%, specificity was 98.82%, and precision was 90% based on the assessment conducted using the confusion matrix [73].

Deep learning algorithms are gaining importance in the prognosis and prediction of diseases using patients’ data [76]. It is pertinent to state that the lack of prompt diagnosis and treatment of anemia can lead to a life-threatening illness [51]. Therefore, several artificial intelligence algorithms have been employed to forecast nutritional anemia cases, especially those related to iron deficiency [35,53]. Each algorithm was observed to be optimized for a certain subset of data, and this means there is a need to develop new processing techniques. The trend was identified in a previous study where the properties of each dataset are unique, and the size was governed by the number of records and variables specific to the dataset [74]. The strategy blends machine and deep learning to improve the identification process. These were observed to be in the form of genetic algorithm (GA), stacked autoencoder (SAE), and convolutional neural network (CNN) methods, which were used to predict the HGB, nutritional or iron deficiency, B12 deficiency, and folate deficiency anemia as well as to examine individuals without the illness [74]. Moreover, a confusion matrix was used to assess the model, and the greatest level of accuracy for the GA-CNN algorithm was recorded to be 98.5%; the F1 score was 98.8%, sensitivity was 98.7%, and precision was 99.00% [74].

Hemoglobin, a protein contained in red blood cells, is important for the transport and storage of oxygen throughout the body [77]. It has been reported to have the ability to preserve its elasticity, spherical form, and stability in healthy individuals [78]. This is the reason it can float above the red blood cells, but its structure does not ameliorate the symptoms of sickle cell disease [22]. The phenomenon is associated with red blood cells that are twisted and blocked with fluid. It is also important to note that blood circulation is hindered by dysfunctional cells. This is dangerous and has the ability to lead to a range of symptoms, including excruciating pain, organ damage, and even heart attacks [49]. It also has the potential to reduce the average human lifespan. Sickle cell disease identified at an early stage can be treated with antibiotics, vitamins, blood transfusions, painkillers, and other medications. However, the manual grading, diagnosis, and cell counts are time-intensive, and this poses a risk of inaccurate data and misclassification because a single sample usually comprises millions of red blood cells. This is the reason the application of data mining techniques is considered effective and efficient in determining the status of sickle cells inside the human body [75]. An example of this is the adoption of a robust and rapid MLP (multilayer perceptron) classification algorithm to separate sickle cell anemia (SCA) patients into three groups, and the method was observed to surpass the constraints of the manual methods. It was discovered that there are three different types of red blood cells, which include normal, sickle, and thalassemic cells [75]; this discovery was followed by the application of the confusion matrix to analyze the performance of the MLP approach. The results obtained using the 1387 datasets gathered between August 2017 and August 2019 showed a correctness score of 96.04% [75] while the 100 most recent datasets obtained from the Thalassemia and Sickle Cell Society (TSCS) in Rajendra Nagar, Hyderabad, Telangana, India [75] from September 2019 to August 2020 showed 99% [75].

## 3. Materials and Methods

### 3.1. Data Collection

This research was conducted using 165 females and 25 males between the ages of 15 and 41 diagnosed with different kinds of anemia. The data used were compiled by the Clinical Pathology Laboratory at Dr. Sardjito General Hospital in Yogyakarta, Indonesia, and the Department of Clinical Pathology and Laboratory Medicine of the Faculty of Medicine, Public Health, and Nursing at Universitas Gadjah Mada. Moreover, a hematological measure was generated from patients with BTT, IDA, HbE, and a combination of BTT and IDA or HbE and IDA. It is important to note that the Medical and Health Research Ethics Committee (MHREC) of the Faculty of Medicine, Public Health, and Nursing at Dr. Sardjito General Hospital, Universitas Gadjah Mada, issued an ethical letter for the conduct of this research, with the identifier KE/FK/1255/EC/2021. The parameters used include the RBC, Hb, HCT, MCV, MCH, and MCHC in addition to RDW. A total of 24 patients were diagnosed with BTT, 41 with HbE, 104 with IDA, and 21 with the combination method. The definitions of several acronyms used during the investigation are presented in Table 2.

### 3.2. Research Flow

**Figure 3:**
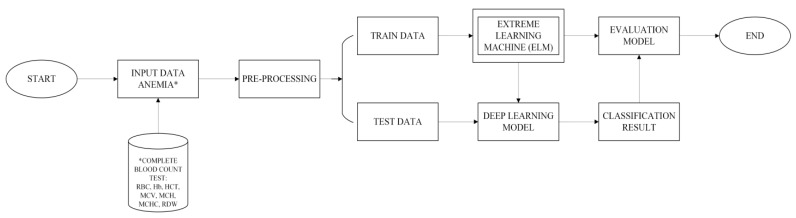
Research flow.

The data derived from the results of a full blood count performed in the laboratory using Advia and Sysmex hematology analyzers produced seven primary characteristics. Moreover, serum ferritin was applied to acquire the gold standard from IDA while hemoglobin electrophoresis was used for BTT and HbE, and the data obtained were examined further and placed in the database based on the flow presented in Figure 3. The seven characteristics previously identified were processed in the database, and the data were labeled by clinical pathology physicians. The data put into the database were preprocessed through cleansing, deletion, the MinMax scaler, and the LabelEncoder. The remaining data were divided into 67%training and 33%testing. Furthermore, the ELM algorithm was used to train the data, which were subsequently applied as the standard to grade the test data. The doctor was involved in the process to provide training courses based on the findings from the laboratory tests. This was followed by the application of the ELM algorithm to classify the data, and its performance was also evaluated. The performance results were further used by the clinical pathology doctor to analyze the data once more to ensure transparency and accountability of the categorization.

### 3.3. Extreme Learning Machine

**Figure 4:**
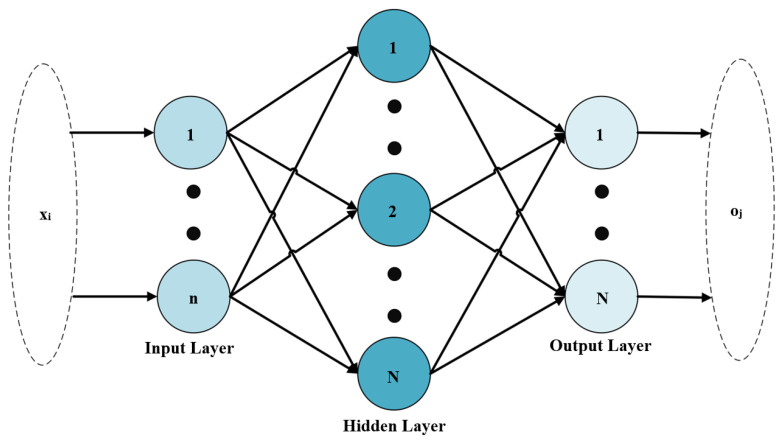
ELM architecture.

The research on the predictive capacities of feedforward neural networks has been mathematically centered on two aspects. The first is the simultaneous estimate of the number of inputs while the second is the estimation within a certain period. Thus, the focus of several studies has been on the feedforward neural networks, as indicated by those conducted on the extensive approximation capabilities of typical multilayer feedforward neural networks [79,80,81,82,83]. Due to their benefits, these networks have been extensively adopted across a variety of commercial sectors over the last few decades. These benefits include the capability to predict complex nonlinear mappings using the available input samples, as well as to provide models for an extraordinarily high number of natural and artificial occurrences, which are considered problematic for standard parametric techniques designed for such events [84]. The single hidden layer feedforward networks, also known as SLFNs, are among the most well-known feedforward neural networks, and their learning and fault tolerance properties have been the topic of discussion in both theoretical and practical studies [85,86,87,88]. The recent development of the extreme learning machine (ELM) neural algorithm for SLFNs [81,88] was used to improve their performance. It is a novel training method that is exceedingly efficient and effective as indicated in Figure 4. The SLFNs were used in this research to analyze anemia data. It is pertinent to note that the behavior of a linear function as a sum of all linear functions in the network is identical to that of a perceptron regardless of the number of layers comprising the neural network [89,90,91]. Thus, a linear function can be described, but there is a possibility of obtaining a nonlinear outcome when an attempt is made to imitate reality. Therefore, a nonlinear activation function was included in the model. It is also pertinent to note that when a network with several layers fails to provide the desired output, the weights and biases need to be modified. The absence of an activation function can cause a change, such as a switch in the neuron signal from 0 to 1, a huge shift, with each neuron feeding a few neurons in the next layer causing a few more neurons to flip. This means minute modifications to the weights and biases used can have a dramatic effect on the end conclusions. Therefore, an activation function was applied to the neuron’s output, and small changes in the function’s weights can lead to moderate changes in the output. Moreover, the sigmoid function receives any number between -infinity and +infinity, but its output is always between 0 and 1. The Adam optimization method is used. This method is the optimal method for these research data in order to obtain optimal performance results. Table 3 explains the mathematical notation used in the ELM formula.

In Equation (2), $x_{i}$ represents the input vector, $o_{j}\;$is the output vector, $\beta_{j}={\left[\beta_{j1},\beta_{j2},\ldots ,\beta_{jm}\right]}^{T}$ indicates the output layer’s density, $w_{j}={\left[w_{j1},w_{j2},\ldots ,w_{jn}\right]}^{T}\;$represents the difference in weight between the input and hidden layers, $b_{j}$ is the function’s threshold, and g(.) is the function of activation. Moreover, the output matrix of hidden layers H and output-hidden layer weights b for the given input-output sample pairs allows the ELM to obtain an output calculated as Hβ=O as indicated in Equation (3),

(2) $$o_{j}=\overset{N}{\underset{j=1}{\sum }}\beta_{j}g\left(\overset{N}{\underset{j=1}{\sum }}w_{j}x_{i}+b_{j}\right),$$

where $w_{j}$ and $b_{j}$ are randomly generated learning parameters of hidden jth nodes, $\beta_{j}\;$are the links connecting hidden jth nodes and output nodes, and g is the sigmoid activation function for ELM.

The $w_{j}\;.\;x_{i}$ part becomes the product of the parts of $w_{j}$ and$\;x_{i}$. Equation (3) is, therefore, presented as follows:

(3) Hβ=O

where

(4) $$H={\left[\begin{array}{ccc} g\left(w_{1}x_{1}+b_{1}\right) & \cdots & g\left(w_{N}x_{1}+b_{N}\right)\\ \vdots & \ddots & \vdots \\ g\left(w_{1}x_{n}+b_{1}\right) & \cdots & g\left(w_{N}x_{n}+b_{N}\right) \end{array}\right]}_{n\times N}$$

(5) $$\beta ={\left[\begin{array}{c} \beta_{1}^{T}\\ \vdots \\ \beta_{N}^{T} \end{array}\right]}_{N\times m},\;\mathrm{and}$$

(6) $$O={\left[\begin{array}{c} O_{1}^{T}\\ \vdots \\ O_{N}^{T} \end{array}\right]}_{N\times m},$$

and H is referred to here as the output matrix of the hidden layer,

(7) $$\hat{\beta }=H^{T}t$$

where $H^{T}$ is the generalized Moore–Penrose inverse of H and t is the target class/data label. Therefore, the output weights were calculated using a mathematical transformation that eliminates the need for a lengthy training phase requiring repeated updates of the network’s parameters through suitable learning parameters such as learning rate and iteration.

It is possible to implement the ELM method in two simple steps, which include the training and testing steps.


**Training Data**
Step 1:Prepare a training data matrix X of N number with features of d.
Step 2:Prepare training data target label t.
Step 3:Determine the number of neurons H in the hidden layer.
Step 4:Create a matrix of initial weight values w of size H×d.
Step 5:Fill w with a random value.
Step 6:Calculate the output hidden layer initialization matrix,           $H_{\mathrm{init}}=X.w^{T}$
(8)Step 7:Calculate the hidden layer output matrix using a sigmoid function.
Step 8:Count $H^{†}$,         $H^{†}={\left(H^{T}.H\right)}^{-1}.H^{T}$
(9)Step 9:Calculate output weight,             $\beta =H^{†}.t$
(10)Step 10:Calculate output value,            O=H.β
(11)
**Testing Data**
Step 1:Prepare a testing data matrix X of N number with features of d.
Step 2:Calculate the output initialization matrix for the hidden layer using step 6.
Step 3:Calculate the output matrix for the hidden layer using step 7.
Step 4:Calculate the output value using step 10.


### 3.4. Blood

All blood cells in the body, as shown in Table 4, are derived from pluripotent stem cells located in bone marrow [52]. It is important to note that one of the basic activities of red blood cells is to transfer oxygen from the lungs to the tissues and also to move carbon dioxide in the opposite direction [78]. Moreover, the platelets, which are vital to hemostasis, circulate for just ten days, but red blood cells have a lifetime of four months [91,92]. It has also been stated that different kinds of phagocytes—including neutrophils, eosinophils, basophils, monocytes, and lymphocytes—comprise white blood cells [93]. The B cells are responsible for the creation of antibodies while the T cells are in charge of immunological responses and defending against viruses and other foreign cells [94]. The white blood cells are present in the blood’s white component to combat illnesses caused by bacteria and fungus. Furthermore, the lymphocytes are responsible for the generation of antibodies. Previous studies have also shown that white blood cells have a relatively lengthy lifetime [95,96].

Red blood cells are the most numerous blood cells [98], and they appear as biconcave discs densely packed with cytoplasm rich in the oxygen-carrying protein hemoglobin on smears of human peripheral blood [68]. They have a clever structure that allows them to perform their primary functions of transporting oxygen from the lungs to the tissues in the body’s periphery and transporting carbon dioxide from the tissues in the body’s periphery to the lungs, where it can be expelled via respiration. This means red blood cells facilitate the exchange of oxygen and carbon dioxide between the lungs and peripheral tissues of the body [99]. They also have an average lifespan of 120 days [93]. Meanwhile, platelets which are also known as thrombocytes are microscopic, fully nucleated, and granular-colored cell fragments. They are usually released by the megakaryocytes in bone marrow [98] and play a key part in the control of hemostasis together with the clotting factors of plasma [99,100]. Platelets have a seven- to ten-day lifespan [101]. It has also been discovered that there are several varieties of white blood cells [94]. These include the granulocytes, which are bone marrow-derived, short-lived cells that look identical on a peripheral smear [101,102,103,104]. Neutrophils, sometimes referred to as polymorphonuclear leukocytes, are the most prevalent kind of white blood cell, which possess between three and five lobes on their nucleus and an abundance of light purple granules in their cytoplasm [105]. They are phagocytes that provide defense against a variety of acute pathogens [105].

Monocytes are the biggest white blood cells, ranging from 12 to 20 μm in diameter [105]. They have a folded or kidney-shaped nucleus and an abundance of light blue cytoplasm with a modest number of extremely tiny granules [106]. Monocytes, like neutrophils, are extremely phagocytic, although they vary from neutrophils in a crucial aspect [107]. They primarily develop into relatively long-lived macrophages capable of recognizing “danger” signals created by infection or tissue damage upon emigration into tissues [108]. Meanwhile, eosinophils with a diameter of 12 to 15 have two nuclear lobes and an abundance of red cytoplasmic granules (as befits the cell named after Eos, goddess of the dawn) [109]. They have a crucial role in some chronic immunological responses, including those linked with worm infections, asthma, and certain forms of allergic reactions [110].

The rarest of the granulocytes is basophil, and its nucleus is enveloped by numerous dark blue cytoplasmic granules [111]. They have a diameter of 12 to 15 μm [110], and many of the circumstances linked with an increase in eosinophil counts are also related to a small rise in basophil numbers [112]. It was also discovered that the mononuclear cells, another kind of white blood cell, lack the segmented nucleus typical of granulocytes [113]. Furthermore, lymphocytes are an essential part of the adaptive immune system [114] and are found to be approximately the same size, 7 to 9 μm in diameter, as a typical red blood cell while at rest and feature a spherical, compact nucleus with minimal cytoplasm [115]. However, the active cells have the potential to grow to a maximum size of 20 μm and also have a small number of granules in addition to the expanded nucleus and copious cytoplasm [115]. Unless the cells are evaluated for the presence of certain lineage-specific markers, it is impossible to tell with absolute certainty whether circulating lymphocytes are B cells, T cells, or natural killer cells. This is due to the fact that the lymphocytes circulating in the blood can be any of these three types [116]. The immune system also has the ability to “remember” the pathogen exposures from many years ago since it has the necessary foundation due to its longevity [117].

### 3.5. Anemia

Anemia is usually defined through the blood hemoglobin level which is below what is considered normal for a person’s age and gender, as indicated in Table 5. The results can vary across labs, but the average values for adult men and women are fewer than 135 g/L and 115 g/L, respectively [118]. The existence of less than 110 g/L for children between the ages of 2 and puberty implies anemia [119], and because newborns have high hemoglobin levels, the minimum acceptable threshold at birth is 140 g/L [120,121]. The World Health Organization classifies individuals as having anemia when their hemoglobin levels fall below 130 g/L for males and 120 g/L for women [122]. This scenario shows that approximately 40% of the world’s population was expected to suffer from anemia in 2019. There is a higher prevalence in females than males of any age, and in children less than five years old. Moreover, the greatest occurrence throughout the globe has been reported in Sub-Saharan South Asia, and Central, West, and East Africa [23]. The primary causes were found to be iron deficiency (hookworms, schistosomiasis), sickle cell disease, thalassemia, malaria, and chronic diseases [123].

**Figure 5:**
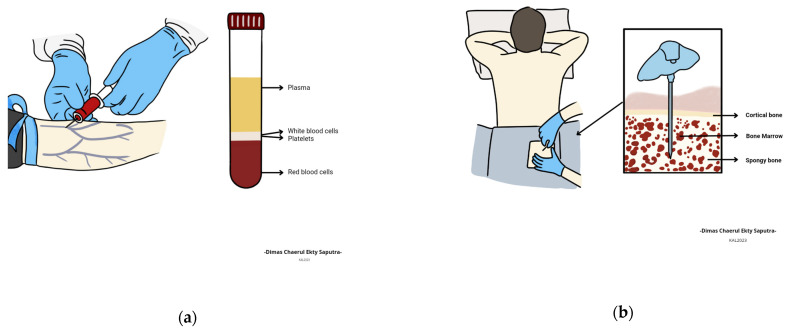
Adapted from Refs. [130] and [131] about complete blood count (CBC) test (**a**) and bone marrow aspirate (**b**). Both tests are usually used to further identify anemia.

Physicians usually inquire about the patient’s medical and family history, conduct a physical examination, and perform some tests including a full blood count to diagnose anemia [124]. More concern is usually placed on the hematocrit and hemoglobin levels, as well as the total number of red blood cells present in the patient’s blood, as indicated in Figure 5a. The natural differences between the quantity of blood components present in males and females are presented in Table 4 [97]. It is important to note that the blood counts can possibly be lower in those engaging in or those who have engaged in significant physical activity, particularly in pregnant women or the elderly [125,126]. Smoking and being at higher altitudes can also increase the number [124,125]. The testing process usually requires analyzing the size and content of red blood cells [127,128] as well as the shape and color deviations. A doctor can also prescribe further tests to establish the underlying reason and occasionally examine a sample of bone marrow to determine the existence of anemia, as indicated in Figure 5b [129].

**Figure 6:**
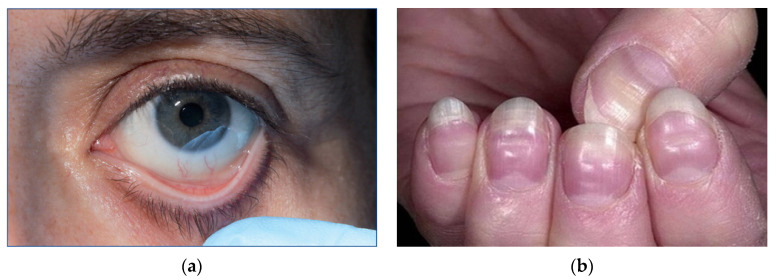
Reproduced with permission from Paul A. H. Moss Victor Hoffbrand, Hoffbrand’s Essential Haematology 7th Edition; published by John Wiley and Sons, 2015 about conjunctival mucosa (**a**) and nail beds (**b**) of two patients with severe anemia (hemoglobin of 60 g/L) [97].

Some patients can exhibit symptoms such as shortness of breath (particularly during physical exercise), weakness, tiredness, palpitations, and headaches [121,122]. Other symptoms—such as heart failure, angina pectoris, intermittent claudication, and disorientation—are more prevalent among the elderly [132,133,134]. Moreover, vision impairment due to retinal hemorrhages can be a serious consequence of anemia, particularly when it develops rapidly [135]. These signals can be classified as either generic or particular. It is also important to note that pallid mucous membranes and nail beds, as shown in Figure 6, are prominent indicators of a hemoglobin concentration below 90 g/L. It is pertinent to state that the color of a person’s skin is not a reliable indicator, but tachycardia, pulse rate, cardiomegaly, and a systolic flow murmur indicate hyperdynamic circulation, particularly at the apex. The symptoms of congestive heart failure can also manifest at any age, but they are more prevalent in older people. Furthermore, certain symptoms are linked to each subtype of anemia, such as koilonychia, sometimes referred to as “spoon nails”, with iron deficiency, jaundice with hemolytic or megaloblastic anemia, foot ulcers with sicklecell and other hemolytic anemias, and skeletal abnormalities with thalassemia major [136]. Koilonychia is usually caused by the deficiency of iron in the body and is classified as a disorder characterized by inwardly curled nails resembling spoons [137]. It is important to note that megaloblastic anemia is the most prevalent kind.

The conjunction of anemic symptoms with severe infection or spontaneous bruising shows the presence of neutropenia or thrombocytopenia, potentially due to bone marrow failure [137]. People with blood hemoglobin levels below the values considered normal for their age and gender are believed to have anemia. Moreover, individual cell size can be used to assess when red blood cells are macrocytic, normocytic, or microcytic. It is also pertinent to state that the cause of anemia can be diagnosed in part by examining the reticulocyte count, the red blood cell shape, and any changes to the white blood cell and/or platelet count [123]. The common clinical manifestations include exertional dyspnea, pale mucous membranes, and tachycardia [138], while the other symptoms associated with some forms of anemia include jaundice and leg ulcers [139]. The aspiration or trephine biopsy of bone marrow can also be used to investigate anemia and a variety of other hematological disorders [140]. It is also possible to conduct specialized examinations such as immunology and cytogenetics on the cells recovered [140].

## 4. Experimental Results

The experiment involved using an ELM model to identify and categorize illness in a dataset of individuals with beta thalassemia trait, iron deficiency anemia, hemoglobin E, and the combinations previously defined. The model parameters utilized are listed in Table 2. The real anemic dataset was used and split into training and testing sets during the experiment, and the classification method applied was evaluated using a Python-written confusion matrix. The process was conducted on an Apple M1 machine with 512GB internal memory and 8GB RAM.

### 4.1. Evaluation Model

The data used were classified into test and training data, and they were both evaluated using the confusion matrix model. This was necessary due to the feasibility of determining the accuracy of classification algorithms using an industry-standard technique. It was discovered that the dataset had five separate classifications, which included the BTT, IDA, HbE, and their combinations. The training data accounted for 67% of the entire data set for the inquiry while test data made up the remaining 33%. The assessment conducted was based on the accuracy, precision, sensitivity, and F1 score of the classification algorithm as listed in Equations (12)–(15). The method of value distribution is highlighted in Table 6.

Table 6 shows that positive data correctly classified by the system are referred to as the “true positive” (TP), negative data correctly identified as negative are referred to as the “true negative” (TN), negative data incorrectly perceived as positive are known as “false negatives” (FN), and “positive” data incorrectly recognized as “positive” are “false positives” (FP).

These values were further used to determine the accuracy, precision, recall, and F1 scores through the following formulas.

(12) $$\mathrm{Accuracy}:\;\frac{\mathrm{TP}+\mathrm{TN}}{\mathrm{TP}+\mathrm{TN}+\mathrm{FP}+\mathrm{FN}}$$

(13) $$\mathrm{Precision}:\;\frac{\mathrm{TP}}{\mathrm{TP}+\mathrm{FP}}$$

(14) $$\mathrm{Sensitivity}:\;\frac{\mathrm{TP}}{\mathrm{TP}+\mathrm{FN}}$$

(15) $$F1\text{-}\mathrm{Score}:\;2\times \frac{\mathrm{Recall}\;\times \;\mathrm{Precision}}{\mathrm{Recall}\;+\;\mathrm{Precision}}$$

### 4.2. Experimental Results of Extreme Learning Machine

The ELM classification model applied to categorize the anemia dataset used a single feedforward network with a hidden layer implementation (SLFNs). This strategy reduced the processing time required for the concealed layer. The usefulness of the model was assessed based on accuracy, precision, sensitivity, and the F1 score in classifying anemic datasets. Table 7 shows the model used in the ELM. The findings of the ELM performance model are presented in Table 8, and it was discovered that it performed best on the four-class anemia dataset, with 99.21% accuracy, 99.30% precision, 98.44% sensitivity, and 98.84% F1 score.

The confusion matrix for the model is presented in Table 9, with each row representing an instance of the prediction class while each column indicates an instance of the actual class. The RF approach use n estimators = 400, max features = auto, and entropy. Although entropy is more sophisticated than the Gini index, entropy provides ideal results. In contrast, the KNN technique employs several experiments, including Euclidean distance to determine the distance between classes, and K = 15, which is derived from the K error rate calculation. Several tests were conducted by employing polynomial kernels, RBF kernels, and linear kernels in the SVM approach. In linear kernels, optimal outcomes were obtained. The ELM model, which is the approach described in this work, employed the sigmoid activation function, with the number of hidden layers (9) modified based on the number of inputs and outputs, followed by gradient descent to optimize the weights.

ELM was used to optimize the classification process for anemic datasets in order to improve the success rate of the approach as indicated in Table 9. The performance index of each class and the recommended strategy with the highest rate of success are, therefore, presented in Table 10. It was discovered that the random forest, k-nearest neighbor, support vector machine, and extreme learning machine techniques provided the most accurate predictions for the beta thalassemia trait and iron deficiency anemia classes. Moreover, the forecasts for the hemoglobin E and the combination classes were rather correct.

## 5. Discussion

It is very dangerous in the field of medicine to erroneously identify healthy individuals with sickness and vice versa due to the possibility of severe repercussions. This has led to an increase in the usage of data mining technologies for a reliable diagnosis. Therefore, this research used a model of an extreme learning machine to reliably detect and diagnose anemia as well as construct a decision support system to aid clinicians in making decisions.

A total of 127 training and 63 test data were employed, and it was discovered that the ELM approach performed much better than RF, KNN, and SVM as indicated by its 99.21% accuracy, 98.44% sensitivity, 99.30% precision, and 98.84% F1 score compared to RF’s 77.01% accuracy, 90.83% precision, 78.40% recall, and 80.99% F1 score as well as KNN’s 65.42%, 59.40%, 62.81%, and 51.74%, respectively. A previous study by [67] used 6935 data with 986 variables and applied two of the five techniques, KNN (92.36%) and RF (94.16%), to classify BTT and IDA into two groups. Another study by [69] predicted the risk of childhood anemia using several machine learning techniques including KNN and RF. The results showed that KNN had a classification accuracy of 61.95%, a sensitivity of 65.85%, and a specificity of 58.20%while RF had 68.5%, 70.7%, and 66.4%, respectively. This means the overall performance of RF was better than KNN in all three aspects. Another research conducted in 2020 [58] showed that RELM had an accuracy of 95.59% when applied to separate 342 patient records into two types of anemia, IDA and BTT. It is important to note that the ELM method was also applied in the research.

Thus, studies have been conducted on the ELM approach, and the concept has progressed to the point where it has shifted from a single hidden layer to a 100-node multilayer hidden layer. This is known as the enhanced improved multilayer extreme learning machine (IML-ELM) with the neural activity occurring both during and after training in the proposed network architecture. Moreover, each layer of the first IML-ELM (IML-ELM1) network was assigned an orthonormal random connection weight while only the very first layer of the second iteration of the IML-ELM contained the random orthonormal connection weights (IML-ELM2). The output weight matrix of the layer was used to calculate the connection weights. The application of the IML-ELM2 assignment method considerably reduced the amount of time required for calculations, and the root mean square error test was observed to have produced 0.627977, 0.104272 (83%), and 0.092685 (85%) [143].

The three studies conducted by [58,67,69] used several machine and deep learning techniques, and RELM was reported to have the highest level of performance with 95.59% in distinguishing two forms of anemia. The outcomes of this experiment conducted using the ELM method have been encouraging, with the anemia classified into four separate subtypes, thereby increasing the diagnostic accuracy to 99.21%, precision to 99.30%, sensitivity to 98.44%, and F1 score to 98.84%.

Table 11 compares the findings of this research with those from previous studies based on the accuracy metric. It is important to note that this research divided the patients into four distinct groups, including BTT, IDA, HbE, and combinations, and the differences between these groups were categorized with a greater degree of precision than previous approaches, as indicated by the 99.21% recorded for each class. Future studies are expected to focus on analyzing the characteristics considered to be the most important components of anemia to ensure an easier diagnosis process for physicians using a more ideal system. There is also the need for a technique to identify and recommend appropriate anemia datasets using deep learning.

## 6. Conclusions

It is difficult to distinguish between BTT, IDA, and HbE, as well as combinations of these three variables, due to the variability of the anemia-afflicted population. The introduction of computer models was observed to have ensured the rapid screening of anemia at a lower cost. This research provided a summary of the findings of the health system analysis as well as the challenges and barriers encountered throughout the globe in treating anemia patients by using a thorough analysis. Therefore, an ELM approach was applied to expedite the identification of different kinds of anemia. The method using 190 data and seven parameters was found to have an accuracy, sensitivity, and precision of 99.21%, 98.44%, and 99.30%, respectively, as well as an F1 score of 98.84% using a confusion matrix. This means it has a high performance and can be applied quickly and at a cheaper cost. The ELM approach is believed to have the capacity to supplement current indices and formulas developed to aid in the operations of healthcare professionals.

## Figures and Tables

**Table 1:** Adapted from Ref. [52] about tools to analyze different types of anemia. Copyright (2015), D. Provan, T. Baglin, I. Dokal, and J. de Vos.

Type	Analyte
Anemia	Blood Hemoglobin
Hemoglobinopathy: Thalassemia Trait; Hemoglobin E; Hemoglobin S	Hemoglobin/Capillary Electrophoresis
Iron Deficiency	Serum Ferritin and Serum Transferrin Receptor (sTfR)
Folate Deficiency	Erythrocyte Folate
Vitamin B12 Deficiency	Serum Cyanocobalamin
Inflammation	Serum C-Reactive Protein (CRP)

**Table 2:** List of laboratory examination abbreviations used in this study.

Full Name	Abbreviation
Red Blood Cell	RBC
Hemoglobin	Hb
Hematocrit	HCT
Mean Corpuscular Volume	MCV
Mean Corpuscular Hemoglobin	MCH
Mean Corpuscular Hemoglobin Concentration	MCHC
Red-Cell Distribution Width	RDW
β-Thalassemia Trait	BTT
Iron Deficiency Anemia	IDA
Hemoglobin E	HbE

**Table 3:** Explanation of mathematical notation.

Variable	Definition
O	Output layers
β	Weight of output layer
g	Activation functions
w	Weight vector between the input and hidden layers
x	Input vector
b	Threshold functions
H	Number of neurons in hidden layers
t	Target class
$H_{\mathrm{init}}$	Output hidden layer initialization matrix
$H^{T}$	H

**Table 4:** Adapted from Ref. [97] about blood cells. Copyright (2016), A. V. Hoffbrand and P. A. H. Moss.

Cell Type	Diameter	Lifespan in Blood	Number of Cells	Function
Red cells 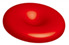	6–8	120 days	Male: $4.5-6.5\times 10^{6}$ Female: 3.9$-5.6\times 10^{6}$	Conveyance of oxygen and carbon dioxide
Platelets 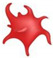	0.5–3.0	10 days	$140-400\times 10^{3}$	Hemostasis
**Phagocytes**				
Neutrophils 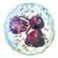	12–15	6–10 h	$1.9-7.6\times 10^{3}$ (48–76%)	Protection against organisms such as bacteria and fungi
Monocytes 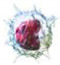	12–20	20–40 h	$0.2-0.8\times 10^{3}$ (2.5–8.5%)	Protection against organisms such as bacteria and fungi
Eosinophils 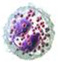	12–15	Days	$0.04-0.44\times 10^{3}$ (<5%)	Protection against parasites
Basophils 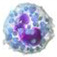	12–15	Days	$0.01-0.1\times 10^{3}$ (<1.5%)	Release histamine for inflammatory responses
Lymphocyte 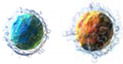 **B** **T**	7–9 (resting) 12–20 (active)	Weeks or years	$1.5-3.5\times 10^{3}$ (18–41%)	**B-cells**: Releases antibodies and assists activation of T-cells. **T-cells**: Protection against viruses; immune function.

**Table 5:** Normal values for blood cells [97].

Type	Males	Females
Hemoglobin (g/L)	135.0–175.0	115.0–155.0
Erythrocytes $\left(\times 10^{2}L\right)$	4.5–6.5	3.9–5.6
Hematocrit (%)	40–52	36–48
Mean Corpuscular Volume (fL)	80–95	
Mean Corpuscular Hemoglobin (pg)	27–34	
Leucocytes	50–150	
Total $\left(\times 10^{9}L\right)$	4.0–11.0	
Neutrophils $\left(\times 10^{9}L\right)$	1.8–7.5	
Monocytes $\left(\times 10^{9}L\right)$	0.2–0.8	
Eosinophils $\left(\times 10^{9}L\right)$	0.04–0.44	
Basophils $\left(\times 10^{9}L\right)$	0.01–0.1	
Lymphocyte $\left(\times 10^{9}L\right)$	1.5–3.5	
Platelets $\left(\times 10^{9}L\right)$	150–400	
Serum Ferritin (μg/L)	40–340	14–150
Serum Vitamin B_12_ (ng/L)	160–925 (20–680 pmol/L)	
Serum Folate (μg/L)	3.0–15.0 (4–30 nmol/L)	
Red Cell Folate (μg/L)	160–640 (360–1460 nmol/L)	

**Table 6:** Adapted from Refs. [139,140,141,142] about confusion matrix.

	Real	True	False
Class Prediction			
True		TP	FN
False		FP	TN

**Table 7:** Extreme learning machine models.

Parameters	Extreme Learning Machine
Target (RMSE)	0.001
Inputs	7
Outputs	4
Hidden layers	1
Training data	128
Testing data	62
Hidden layer neurons	9
Output layer neurons	4
Activation function	Sigmoid

**Table 8:** Performance results of the ELM model.

Split Data	Model	Accuracy (%)	Precision (%)	Sensitivity (%)	F1 Score (%)
67% (128 Data) Train–33% (62 Data) Test	Extreme Learning Machine	99.21	99.30	98.44	98.84

**Table 9:** Confusion matrix results from the proposed model compared to other methods.

Model	Classes	BTT	IDA	HbE	Combination
Random Forest	BTT	**6**	0	0	0
	IDA	0	**13**	4	0
	HbE	1	0	**34**	0
	Combination	0	1	2	**2**
K-Nearest Neighbor	BTT	**5**	1	0	0
	IDA	0	**13**	4	0
	HbE	1	2	**32**	0
	Combination	0	2	3	**0**
Support Vector Machine	BTT	**6**	1	0	4
	IDA	0	**13**	2	1
	HbE	1	1	**27**	5
	Combination	0	0	2	**0**
**Extreme Learning Machine**	BTT	**5**	0	0	0
	IDA	0	**15**	1	0
	HbE	0	0	**35**	0
	Combination	0	0	0	**7**

**Table 10:** Index of performance results for each class in each method.

Model	Classes	Accuracy (%)	Precision (%)	Sensitivity (%)	F1 Score (%)
Random Forest	BTT	100	85.71	100	92.30
	IDA	88.57	92.86	76.47	83.87
	HbE	96.55	85	97.14	90.66
	Combination	90.91	100	40	57.14
K-Nearest Neighbor	BTT	89.70	83.34	83.34	83.34
	IDA	87.88	72.23	76.47	37.14
	HbE	89.28	82.05	91.43	86.49
	Combination	85.29	0	0	0
Support Vector Machine	BTT	96.61	85.71	54.54	66.61
	IDA	93.44	86.67	81.25	83.87
	HbE	82.54	87.10	79.41	83.08
	Combination	80.95	0	0	0
**Extreme Learning Machine**	BTT	100	100	100	100
	IDA	98.44	100	93.75	96.77
	HbE	98.41	97.22	100	98.59
	Combination	100	100	100	100

**Table 11:** Competitive results from other methods.

Authors	Year	Data Size	Number of Classes	Method	Accuracy (%)
Meena et al. [143]	2019	259,627	4	**Decision Tree**	97.35
Sow et al. [144]	2019	6935	4	**ANN**, SVM, RF, and NB	94.74
Laengsri et al. [67]	2019	186	2	**DT**, KNN, RF, ANN, and SVM	98.03
Ayyildiz and Tuncer [35]	2019	342	2	**SVM** and KNN	96.20
Kilicarslan et al. [74]	2020	15,300	5	**GA-CNN** and GA-SAE	98.50
Çil et al. [58]	2020	342	2	ELM, **RELM**, SVM, and KNN	95.59
Tyas et al. [145]	2020	7108	9	**Multilayer Perceptron**	93.77
De and Chakraborty [146]	2021	200	2	LR, RF, NB, MLP, DT, and **KNN**	92.00
Fu Yi-Kai et al. [147]	2021	350	3	**Support Vector Machine**	76.00
Dejene et al. [148]	2022	11,174	4	RF, Extreme Gradient Boosting, and **Cat Boost**	97.56
Memmolo et al. [149]	2022	1000	2	DT, DA, NB, **SVM**, KNN, and Ensemble Learning	84.30
Memmolo et al. [149]	2022	1000	5	DT, DA, NB, **SVM**, KNN, and Ensemble Learning	69.50
Islam et al. [30]	2022	3020	2	LR, LDA, KNN, SVM, QDA, NN, CART, and **RF**	81.29
**Proposed Model**	**2023**	**190**	**4**	**ELM**	**99.21**

## Data Availability

Not applicable.
